# Supplementary material for: Giant dielectric tunability in ferroelectric ceramics with ultralow loss by ion substitution design
Source: Nat Commun. 2024 May 4;15:3754. doi: 10.1038/s41467-024-48264-7 (PMC11069505; doi:10.1038/s41467-024-48264-7)
Supplement: Supplementary file 1 — Supplementary Information [file 41467_2024_48264_MOESM1_ESM.pdf]

Supplementary Information

Giant dielectric tunability in ferroelectric ceramics with ultralow loss by ion substitution design

Authors:

Ruitao Li[1], Diming Xu[1]*, Chao Du[1], Qianqian Ma[2], Feng Zhang[3], Xu Liang[2]*, Dawei Wang[3]*, Zhongqi Shi[3], Wenfeng Liu[4], and Di Zhou[1]*

Affiliations:

[1]Electronic Materials Research Laboratory & Multifunctional Materials and Structures, Key Laboratory of the Ministry of Education & International Center for Dielectric Research, School of Electronic Science and Engineering, Xi'an Jiaotong University, Xi’an 710049, China.

[2]State Key Laboratory for Strength and Vibration of Mechanical Structures, Xi’an Jiaotong University, Xi’an 710049, China.

[3]School of Microelectronics & State Key Laboratory for Mechanical Behavior of Materials, Xi’an Jiaotong University, Xi’an 710049, China.

[4]State Key Laboratory of Electrical Insulation and Power Equipment, Xi'an Jiaotong University, Xi'an, 710049, China.

*Corresponding authors. Email:

diming.xu@xjtu.edu.cn (D. X.); xliang226@xjtu.edu.cn (X. L.);

Dawei.wang@xjtu.edu.cn (D. W.); zhoudi1220@gmail.com (D. Z.).

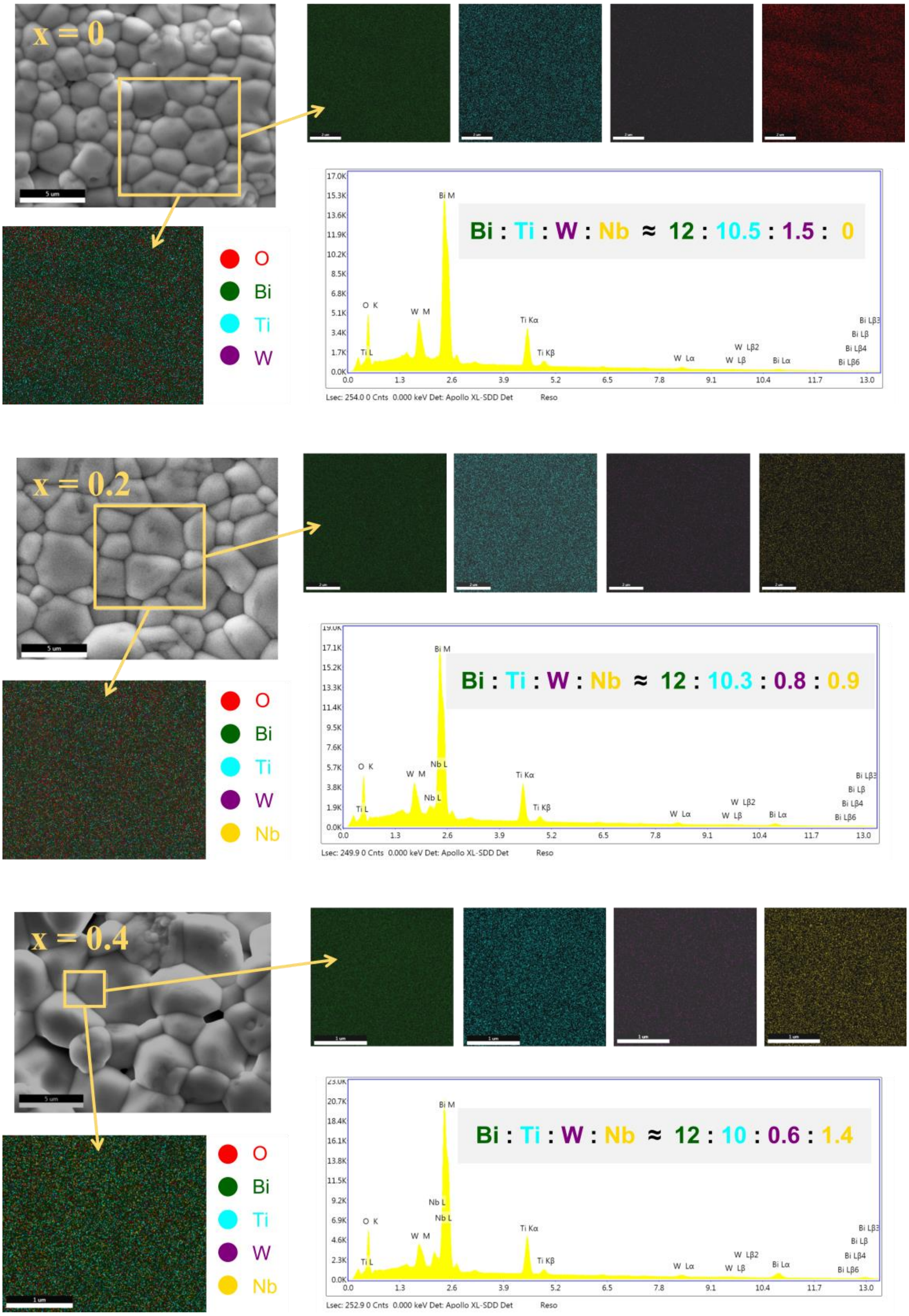

x = 0
5 um
O
Bi
Ti
W
Bi : Ti : W : Nb ≈ 12 : 10.5 : 1.5 : 0
Bi M
O K
W M
Ti Kα
Ti L
Ti Kβ
W Lα
W Lβ2
W Lβ
Bi Lα
Bi Lβ3
Bi Lβ
Bi Lβ4
Bi Lβ6
Lsec: 254.0 0 Cnts 0.000 keV Det: Apollo XL-SDD Det Reso
x = 0.2
5 um
O
Bi
Ti
W
Nb
Bi : Ti : W : Nb ≈ 12 : 10.3 : 0.8 : 0.9
Bi M
Nb L
O K
W M
Ti Kα
Ti L
Ti Kβ
W Lα
W Lβ2
W Lβ
Bi Lα
Bi Lβ3
Bi Lβ
Bi Lβ4
Bi Lβ6
Lsec: 249.9 0 Cnts 0.000 keV Det: Apollo XL-SDD Det Reso
x = 0.4
5 um
1 um
O
Bi
Ti
W
Nb
Bi : Ti : W : Nb ≈ 12 : 10 : 0.6 : 1.4
Bi M
Nb L
O K
W M
Ti Kα
Ti L
Ti Kβ
W Lα
W Lβ2
W Lβ
Bi Lα
Bi Lβ3
Bi Lβ
Bi Lβ4
Bi Lβ6
Lsec: 252.9 0 Cnts 0.000 keV Det: Apollo XL-SDD Det Reso

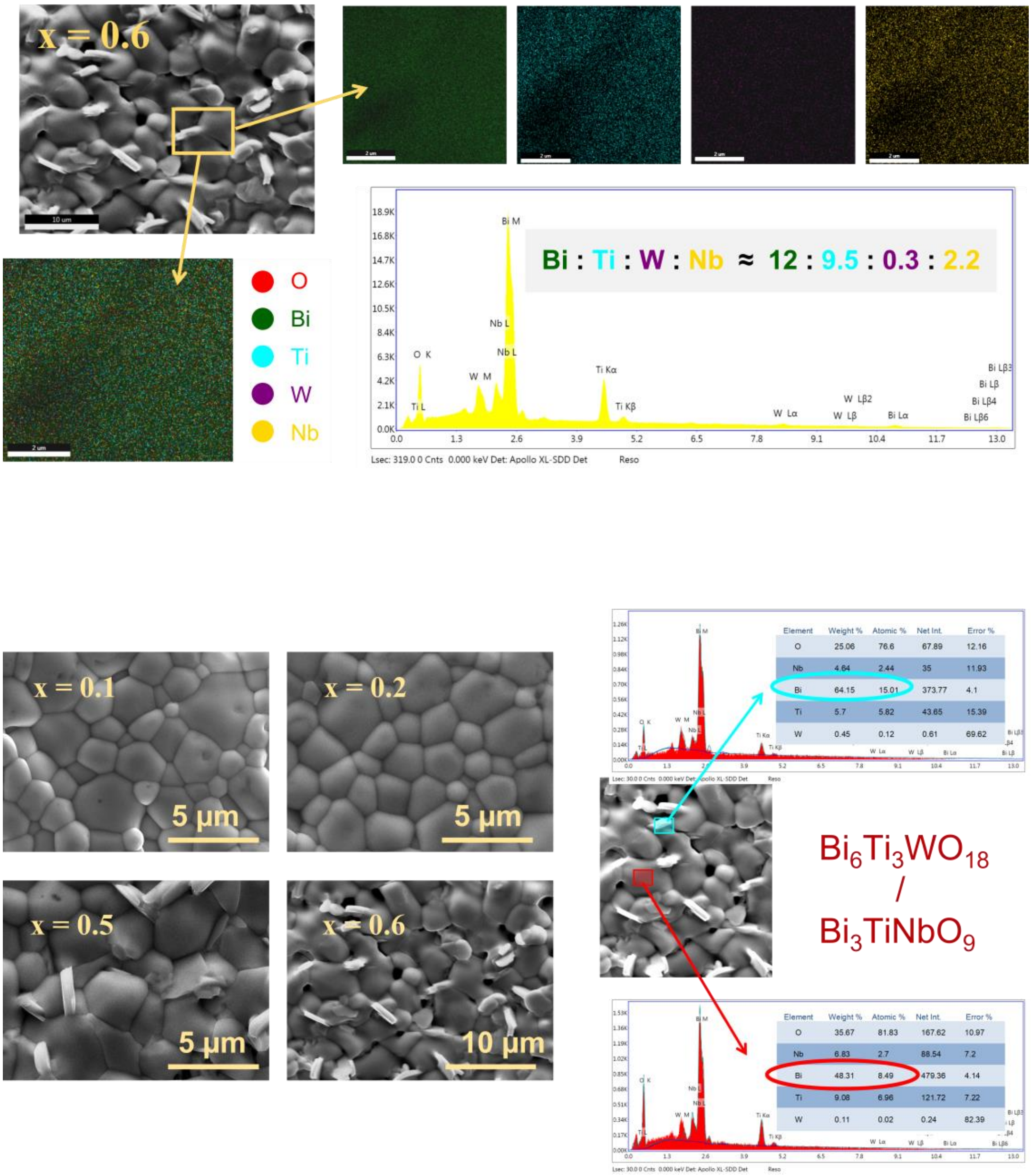

Supplementary Fig. 1. Energy dispersive X-ray spectrometry (EDS) analysis of (1-x)$Bi_6Ti_5WO_{22}$ - $x Bi_6Ti_4Nb_2O_{22}$ ($0 \leq x \leq 0.6$) solid solution ceramics (BTW-BTN). Elemental distribution verifies the successful substitution of Nb^{5+} ions. Elemental analysis of distinct phase structures confirms the composition of the second phase.

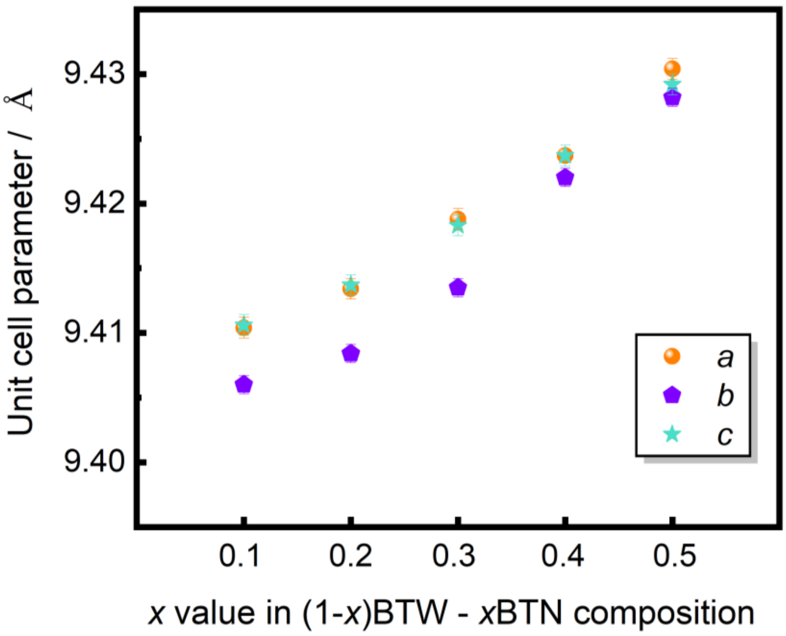
Unit cell parameter / Å
9.43
9.42
9.41
9.40
0.1
0.2
0.3
0.4
0.5
x value in (1-x)BTW - xBTN composition
a
b
c

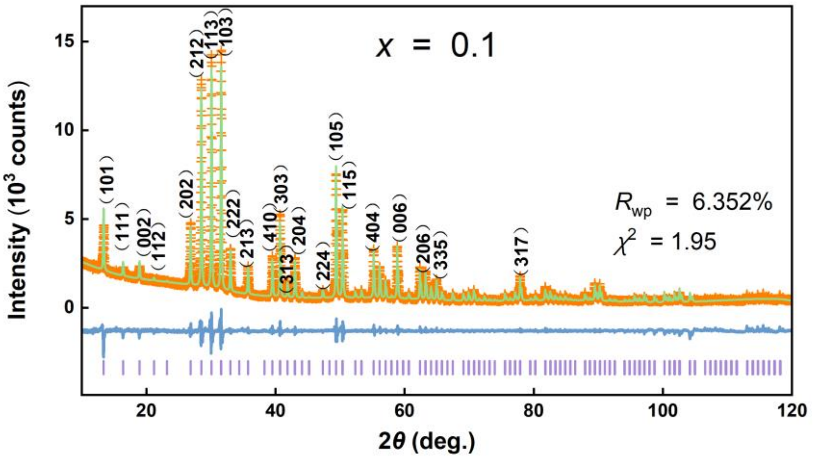
x = 0.1
Intensity (10³ counts)
15
10
5
0
(101)
(111)
(002)
(112)
(202)
(212)
(113)
(103)
(222)
(213)
(410)
(313)
(303)
(204)
(224)
(105)
(115)
(404)
(006)
(206)
(335)
(317)
Rwp = 6.352%
χ2 = 1.95
20
40
60
80
100
120
2θ (deg.)

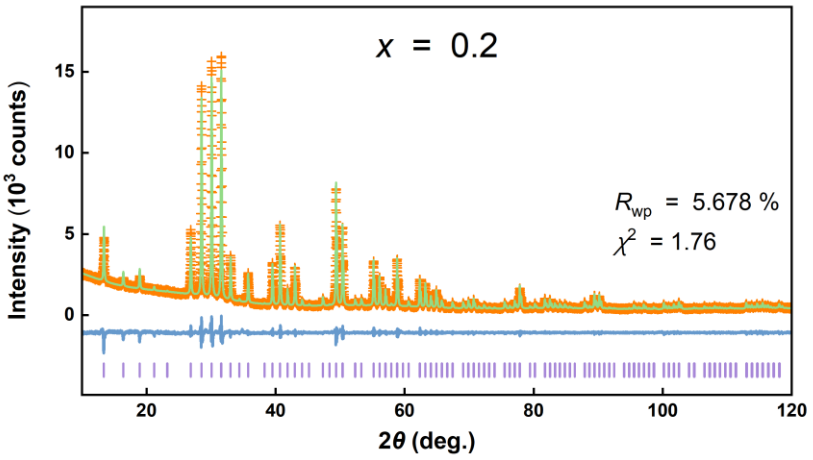
x = 0.2
Intensity (10³ counts)
15
10
5
0
Rwp = 5.678 %
χ2 = 1.76
20
40
60
80
100
120
2θ (deg.)

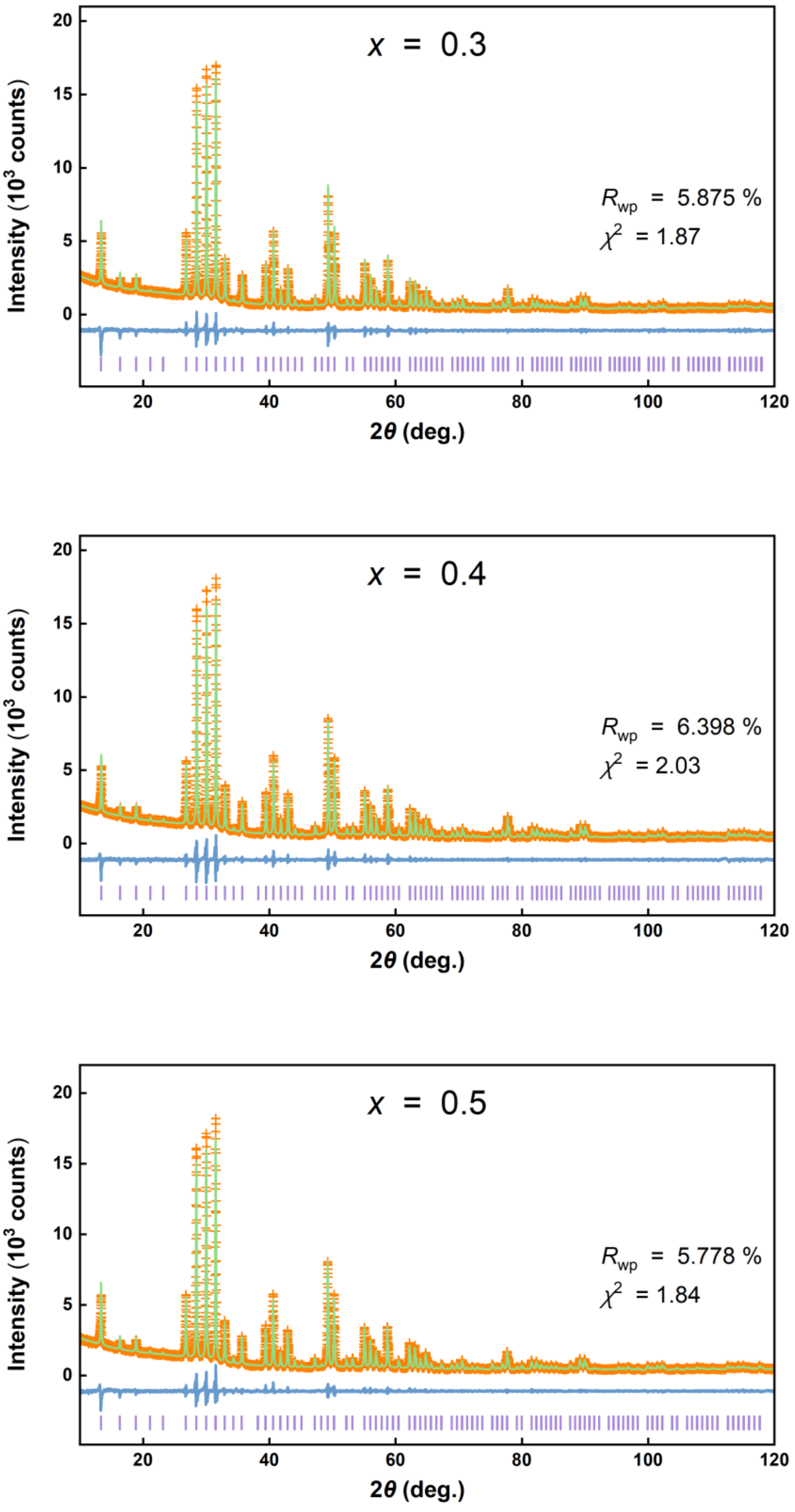

Supplementary Fig. 2. X-ray diffraction refinement results of (1-*x*)BTW - *x*BTN. With the substitution of Nb^{5+} ions, the lattice constant continually increases.

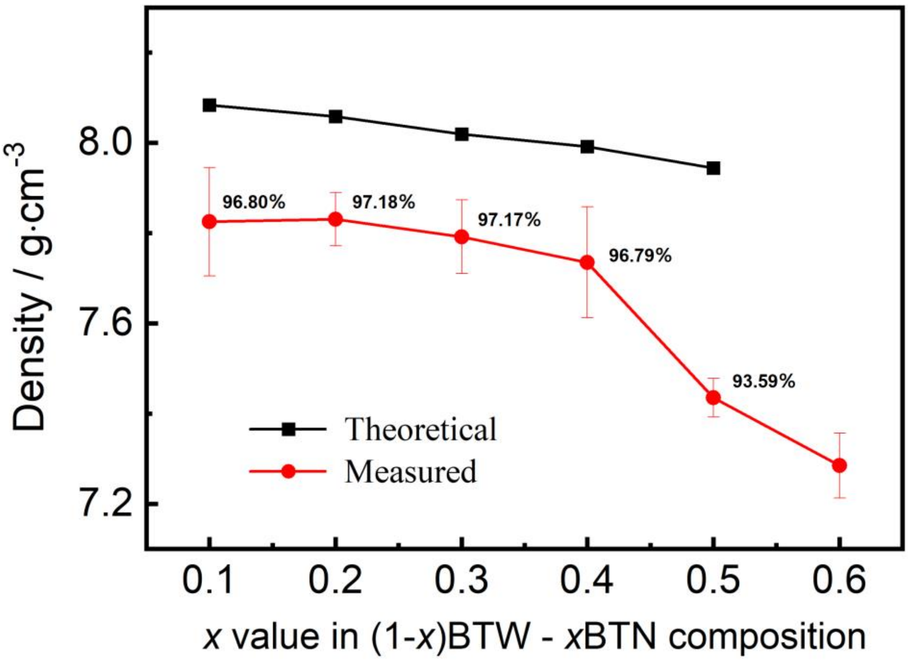

Supplementary Fig. 3. Theoretical and measured density of (1-*x*)BTW - *x*BTN.

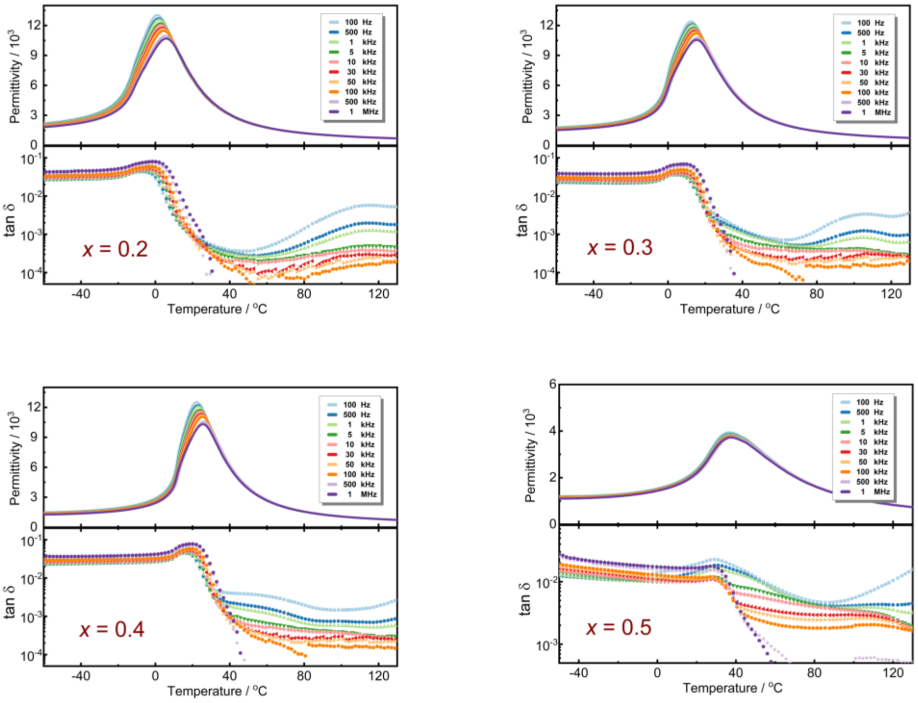

Supplementary Fig. 4. Temperature-dependent dielectric spectra of (1-*x*)BTW - *x*BTN.

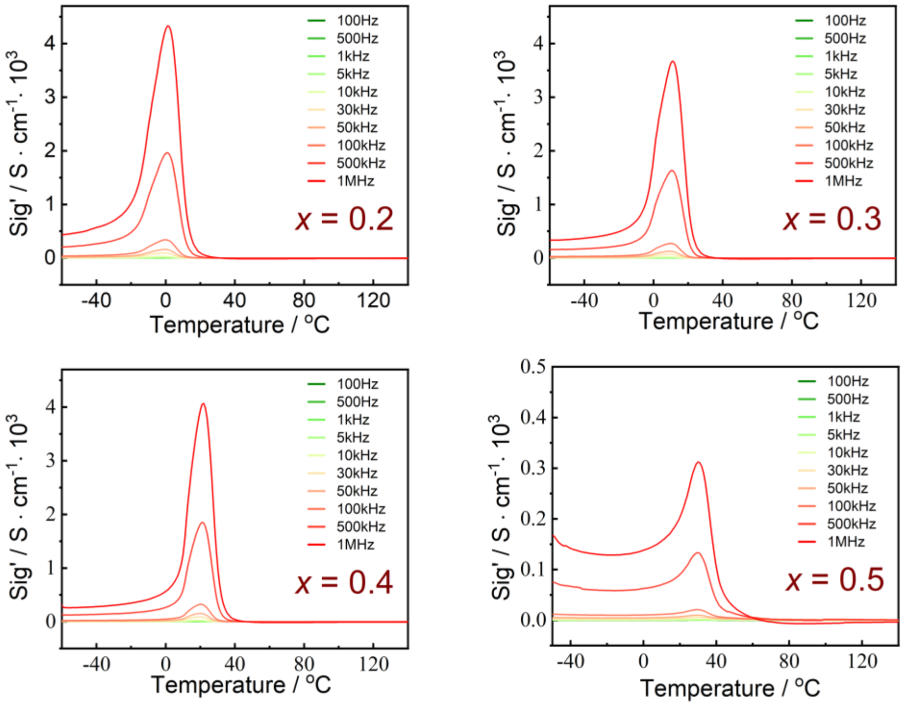

Supplementary Fig. 5. Temperature-dependent conductivity spectra of (1-*x*)BTW - *x*BTN.

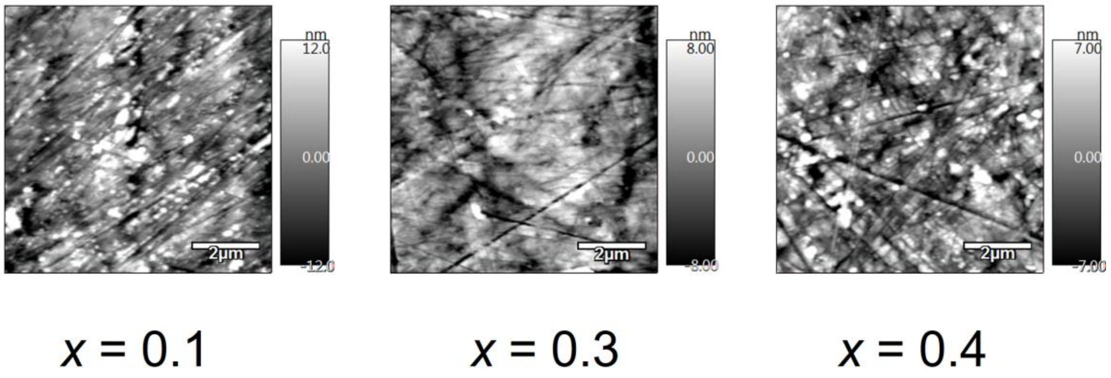

Supplementary Fig. 6. Surface morphology of the samples subjected to atomic force microscopy (AFM) testing.

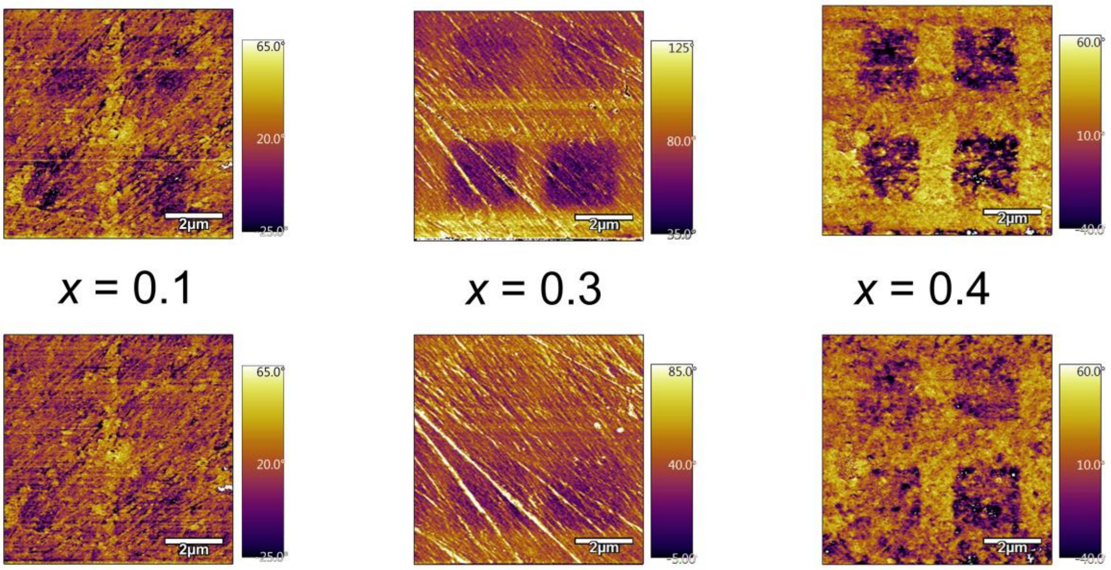

Supplementary Fig. 7. Phase variations of samples subjected to atomic force microscopy (AFM) testing. The images above were captured immediately after applying voltage, while the images below represent the results obtained after a 20-minute interval before testing. The trends in results are analogous to those observed in amplitude.

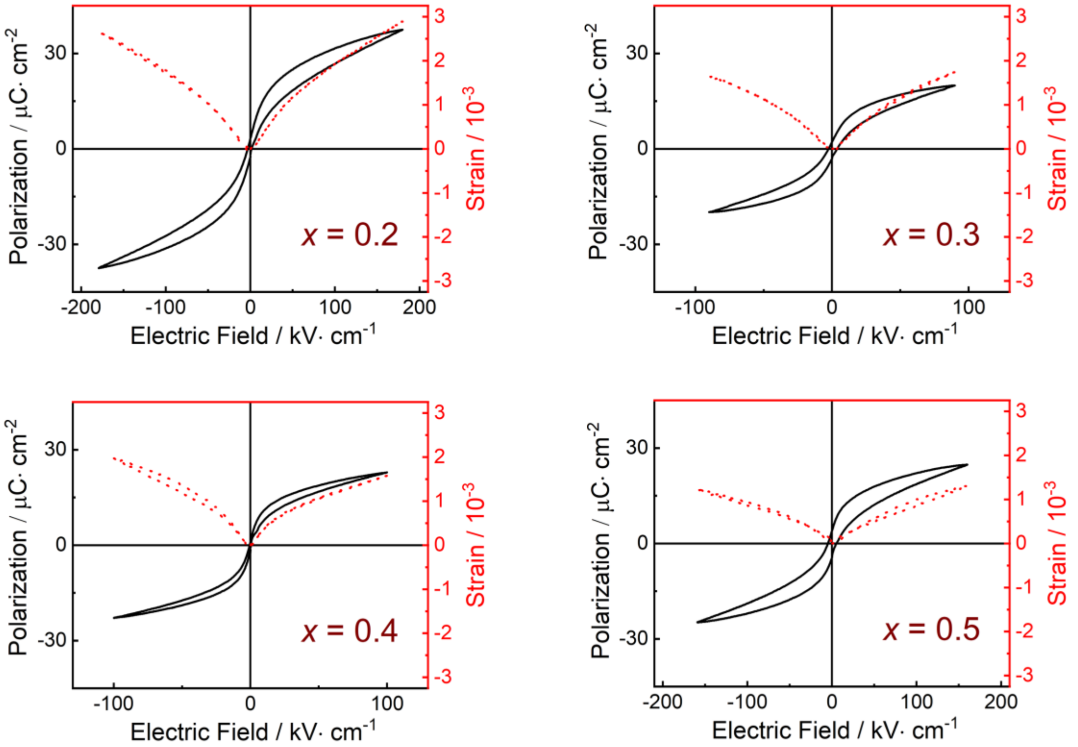

Supplementary Fig. 8. Polarization-electric field hysteresis loops of (1-*x*)BTW - *x*BTN.

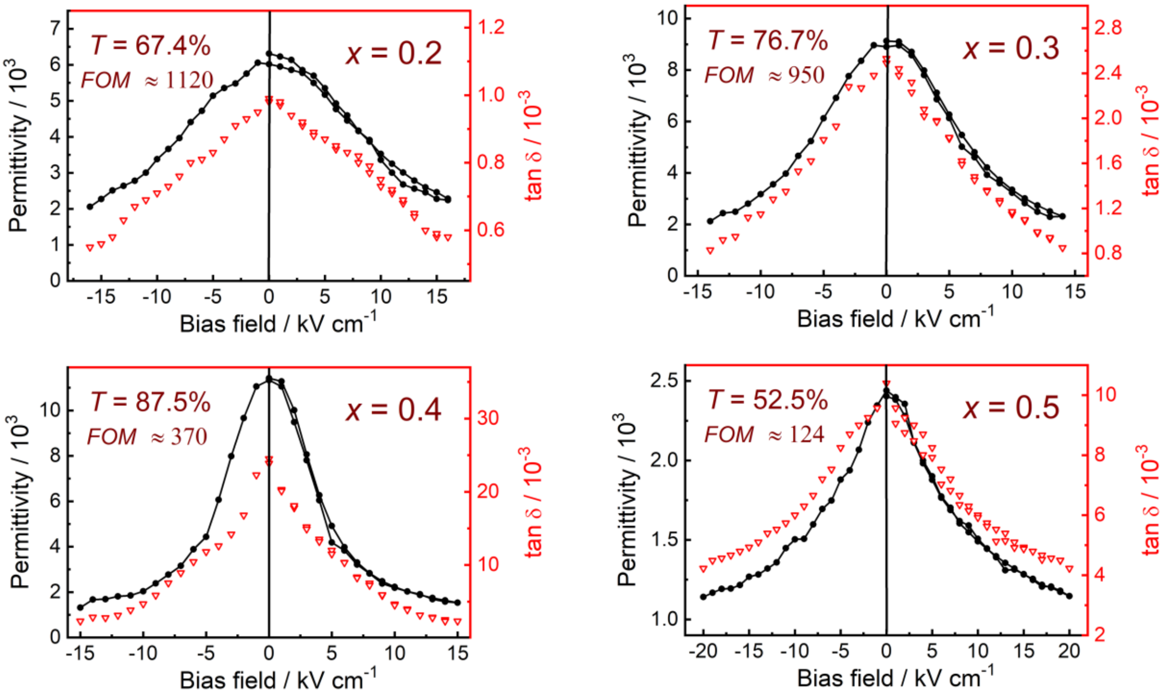

Supplementary Fig. 9. Dielectric tunability of (1-*x*)BTW - *x*BTN.

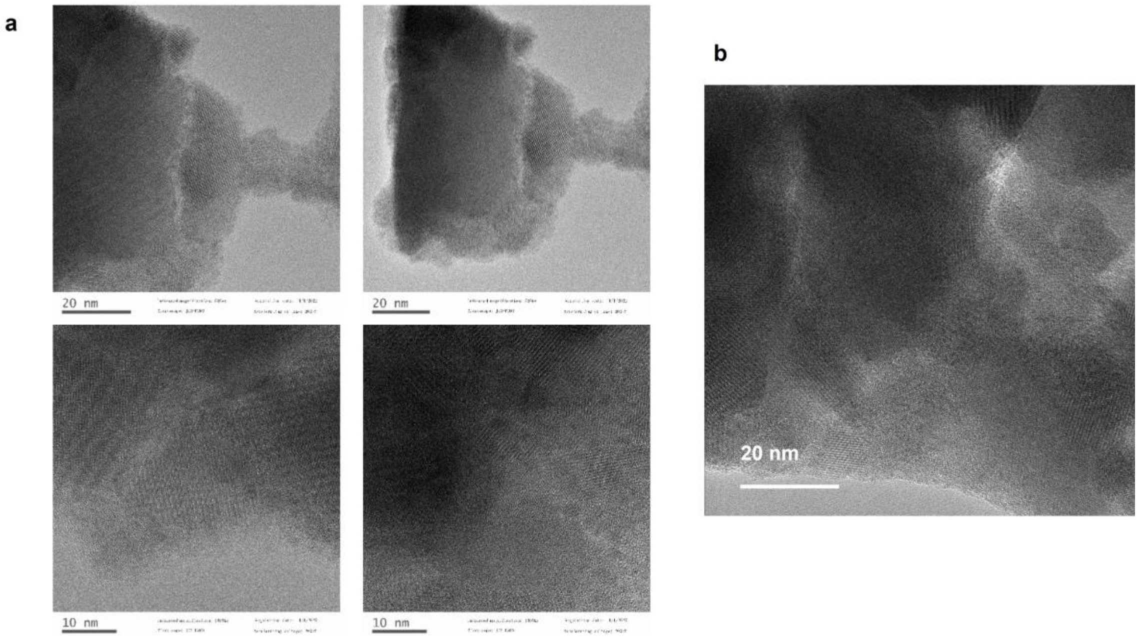

Supplementary Fig. 10. High-angle annular dark-field scanning transmission electron microscopy images of (**a**) $0.6Bi_6Ti_5WO_{22}$ - $0.4Bi_6Ti_4Nb_2O_{22}$, (**b**) pure $Bi_6Ti_5WO_{22}$.

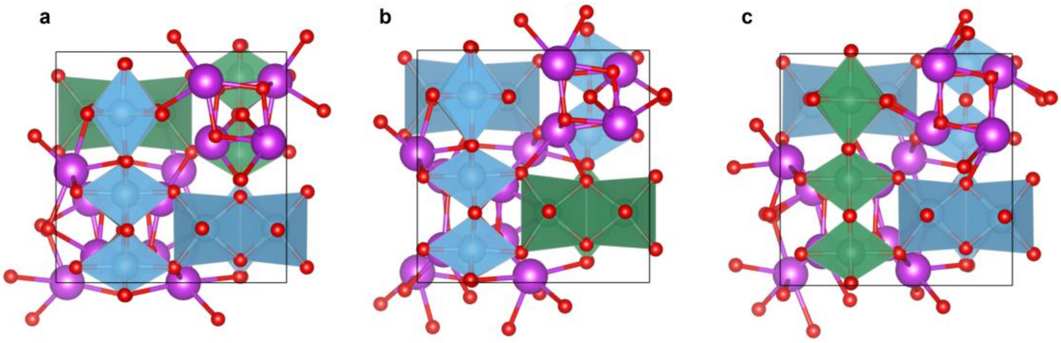

Supplementary Fig. 11. Crystal structure of BTN(3) along (**a**) *a* axis, (**b**) *b* axis, (**c**) *c* axis.

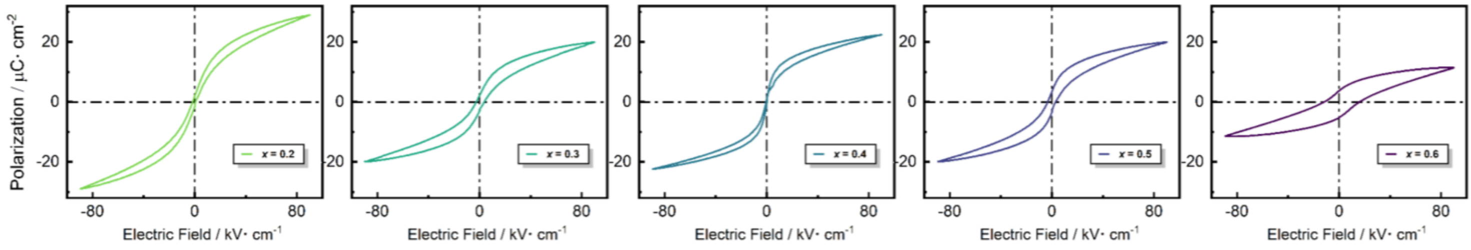

Supplementary Fig. 12. Polarization-electric field hysteresis loops of (1-*x*)BTW - *x*BTN for each component under the same electric field.

Supplementary Table 1. Temperature-dependent neutron powder diffraction refinement results and crystal information of $0.6Bi_6Ti_5WO_{22}$ - $0.4Bi_6Ti_4Nb_2O_{22}$ at 25 ºC.

Refinement Results 25℃					
Space group			*Pnn*2 (No.34)		
a / Å			9.4387(11)		
b / Å			9.4283(8)		
c / Å			9.4360(6)		
V / $Å^3$			839.59(5)		
R_{wp}^{NPD}			5.32%		
χ^2			2.62		
Atomic Parameters					
Atom	*x*	*y*	*z*	Occupancy	$U_{iso}/10^3$ $Å^3$
Bi1	0.1358	0.1454	0.0943		44.7
Bi2	0.3522	0.3677	0.3491		27.1
Bi3	0.2403	0.2570	0.7369		23.0
Ti1	0.5060	0.3482	-0.011	0.5026	23.1
Ti2	0.0	0.5	0.3519		28.3
Ti3	0.0	0.5	0.6421	0.9571	11.9
Ti4	0.3549	-0.0143	0.4393	0.8188	27.0
W1	0.5062	0.3482	-0.0110	0.2786	23.1
W2	0.0	0.5	0.6421	0.0429	11.9
Nb1	0.5060	0.3482	-0.0110	0.2188	23.1
Nb2	0.3548	-0.01435	0.4393	0.1812	27.0
O1	0.5952	0.5941	0.5661		6.2
O2	0.3926	0.6137	0.3589		3.2
O3	0.1237	0.5112	0.4961		3.8
O4	0.5	0.5	0.1063		0.7
O5	0.5	0.5	0.8263		4.7
O6	-0.0084	0.2845	0.3238		2.8
O7	-0.0002	0.2892	0.6413		3.0
O8	0.1594	0.5095	0.2047		2.7
O9	0.1615	0.4998	0.7935		16.4
O10	0.2977	0.3502	-0.0121		10.6
O11	0.2860	0.6550	-0.0060		12.5
O12	0.0075	0.3654	-0.0068		17.7

Supplementary Table 2. Temperature-dependent neutron powder diffraction refinement results and crystal information of $0.6Bi_6Ti_5WO_{22}$ - $0.4Bi_6Ti_4Nb_2O_{22}$ at 50 °C.

Refinement Results 50 °C					
Space group	*Pnn*2 (No.34)				
a / Å	9.4416(10)				
b / Å	9.4250(7)				
c / Å	9.4355(6)				
V / $Å^3$	839.64(5)				
R_{wp}^{NPD}	5.49%				
χ^2	2.86				
Atomic Parameters					
Atom	*x*	*y*	*z*	Occupancy	$U_{iso}/10^3$ $Å^3$
Bi1	0.1400	0.1431	0.0941		45.4
Bi2	0.3584	0.3641	0.3484		29.2
Bi3	0.2422	0.2552	0.7346		23.8
Ti1	0.4985	0.3461	-0.0180	0.5026	23.1
Ti2	0.0	0.5	0.3417		28.3
Ti3	0.0	0.5	0.6421	0.9571	11.9
Ti4	0.3594	-0.0038	0.4389	0.8188	27.0
W1	0.4985	0.3461	-0.0180	0.2786	23.1
W2	0.0	0.5	0.6421	0.0429	11.9
Nb1	0.4985	0.3461	-0.0180	0.2188	23.1
Nb2	0.3594	-0.0038	0.4389	0.1812	27.0
O1	0.5937	0.5971	0.5685		8.5
O2	0.3892	0.6117	0.3585		5.3
O3	0.1235	0.5080	0.4947		7.2
O4	0.5	0.5	0.1067		2.7
O5	0.5	0.5	0.8294		7.1
O6	-0.0117	0.2863	0.3207		3.4
O7	-0.0024	0.2903	0.6367		4.0
O8	0.1605	0.5094	0.2050		3.4
O9	0.1568	0.4997	0.7886		17.1
O10	0.3006	0.3464	-0.0133		12.7
O11	0.2886	0.6577	-0.0086		13.2
O12	0.0074	0.3642	-0.0095		18.5

Supplementary Table 3. Temperature-dependent neutron powder diffraction refinement results and crystal information of $0.6Bi_6Ti_5WO_{22}$ - $0.4Bi_6Ti_4Nb_2O_{22}$ at 100 °C.

Refinement Results 100 °C					
Space group		*Pnn*2 (No.34)			
a / Å		9.4419(12)			
b / Å		9.4274(7)			
c / Å		9.4377(7)			
V / Å^3		840.07(5)			
R_{wp}^{NPD}		5.47%			
χ^2		2.82			
**Atomic Parameters**					
Atom	*x*	*y*	*z*	Occupancy	$U_{iso}/10^3$ Å^3
Bi1	0.1470	0.1380	0.0890		47.6
Bi2	0.3590	0.3635	0.3400		30.5
Bi3	0.2426	0.2563	0.7290		24.5
Ti1	0.5024	0.3365	-0.0131	0.5026	24.0
Ti2	0.0	0.5	0.3400		29.2
Ti3	0.0	0.5	0.6443	0.9571	12.8
Ti4	0.3543	-0.0054	0.4378	0.8188	27.1
W1	0.5024	0.3365	-0.0131	0.2786	24.0
W2	0.0	0.5	0.6443	0.0429	12.8
Nb1	0.5024	0.3365	-0.0131	0.2188	24.0
Nb2	0.3543	-0.0054	0.4378	0.1812	27.1
O1	0.5950	0.5964	0.5600		8.9
O2	0.3920	0.6099	0.3530		6.1
O3	0.1274	0.5095	0.4850		8.2
O4	0.5	0.5	0.1030		4.0
O5	0.5	0.5	0.8230		8.1
O6	-0.0030	0.2867	0.3150		4.1
O7	-0.0000	0.2896	0.6310		4.7
O8	0.1610	0.5080	0.1980		3.9
O9	0.1600	0.4985	0.7790		17.9
O10	0.2960	0.3500	-0.0210		13.2
O11	0.2860	0.6573	-0.0100		13.7
O12	0.0060	0.3688	-0.0120		19.0

Supplementary Table 4. Temperature-dependent neutron powder diffraction refinement results and crystal information of $0.6Bi_6Ti_5WO_{22}$ - $0.4Bi_6Ti_4Nb_2O_{22}$ at 200 °C.

Refinement Results 200 °C					
Space group	*Pnn*2 (No.34)				
a / Å	9.4485(13)				
b / Å	9.4351(8)				
c / Å	9.4449(7)				
V / Å^3	842.00(5)				
R_{wp}^{NPD}	5.44%				
χ^2	2.77				
Atomic Parameters					
Atom	*x*	*y*	*z*	Occupancy	$U_{iso}/10^3$ Å^3
Bi1	0.1407	0.1366	0.0914		48.6
Bi2	0.3564	0.3661	0.3367		32.1
Bi3	0.2452	0.2574	0.7305		26.1
Ti1	0.5050	0.3397	-0.0161	0.5026	24.5
Ti2	0.0	0.5	0.3371		30.1
Ti3	0.0	0.5	0.6381	0.9571	14.6
Ti4	0.3433	-0.0073	0.4359	0.8188	29.1
W1	0.5050	0.3397	-0.0161	0.2786	24.5
W2	0.0	0.5	0.6381	0.0429	14.6
Nb1	0.5050	0.3397	-0.0161	0.2188	24.5
Nb2	0.3433	-0.0073	0.4359	0.1812	29.1
O1	0.6032	0.5935	0.5593		9.7
O2	0.3940	0.6134	0.3546		7.3
O3	0.1294	0.5101	0.4847		9.0
O4	0.5	0.5	0.0978		4.9
O5	0.5	0.5	0.8252		8.8
O6	-0.0029	0.2863	0.3167		5.0
O7	-0.0014	0.2888	0.6314		5.2
O8	0.1612	0.5088	0.1979		4.6
O9	0.1584	0.5002	0.7821		18.1
O10	0.2937	0.3494	-0.0236		14.0
O11	0.2896	0.6568	-0.0107		14.0
O12	0.0055	0.3656	-0.0124		19.3
